# Variety-driven rhizosphere microbiome bestows differential salt tolerance to alfalfa for coping with salinity stress

**DOI:** 10.3389/fpls.2023.1324333

**Published:** 2023-12-11

**Authors:** Wenqiang Fan, Yanzi Xiao, Jiaqi Dong, Jing Xing, Fang Tang, Fengling Shi

**Affiliations:** ^1^ Key Laboratory of Grassland Resources of the Ministry of Education and Key Laboratory of Forage Cultivation, Processing and High-Efficiency Utilization of the Ministry of Agriculture, College of Grassland, Resources and Environment, Inner Mongolia Agricultural University, Hohhot, China; ^2^ College of Agriculture and Forestry, Hulunbuir University, Hulunber, China

**Keywords:** salinity stress, alfalfa (*Medicago sativa* L.), amplicon sequencing, rhizosphere microbiome, plant-microbe interactions

## Abstract

Soil salinization is a global environmental issue and a significant abiotic stress that threatens crop production. Root-associated rhizosphere microbiota play a pivotal role in enhancing plant tolerance to abiotic stresses. However, limited information is available concerning the specific variations in rhizosphere microbiota driven by different plant genotypes (varieties) in response to varying levels of salinity stress. In this study, we compared the growth performance of three alfalfa varieties with varying salt tolerance levels in soils with different degrees of salinization. High-throughput 16S rRNA and ITS sequencing were employed to analyze the rhizosphere microbial communities. Undoubtedly, the increasing salinity significantly inhibited alfalfa growth and reduced rhizosphere microbial diversity. However, intriguingly, salt-tolerant varieties exhibited relatively lower susceptibility to salinity, maintaining more stable rhizosphere bacterial community structure, whereas the reverse was observed for salt-sensitive varieties. *Bacillus* emerged as the dominant species in alfalfa's adaptation to salinity stress, constituting 21.20% of the shared bacterial genera among the three varieties. The higher abundance of *Bacillus*, *Ensifer*, and *Pseudomonas* in the rhizosphere of salt-tolerant alfalfa varieties is crucial in determining their elevated salt tolerance. As salinity levels increased, salt-sensitive varieties gradually accumulated a substantial population of pathogenic fungi, such as *Fusarium* and *Rhizoctonia*. Furthermore, rhizosphere bacteria of salt-tolerant varieties exhibited increased activity in various metabolic pathways, including biosynthesis of secondary metabolites, carbon metabolism, and biosynthesis of amino acids. It is suggested that salt-tolerant alfalfa varieties can provide more carbon sources to the rhizosphere, enriching more effective plant growth-promoting bacteria (PGPB) such as *Pseudomonas* to mitigate salinity stress. In conclusion, our results highlight the variety-mediated enrichment of rhizosphere microbiota in response to salinity stress, confirming that the high-abundance enrichment of specific dominant rhizosphere microbes and their vital roles play a significant role in conferring high salt adaptability to these varieties.

## Introduction

Soil salinization is an increasingly serious global ecological and environmental problem, threatening agricultural production and ecological security worldwide (Zhou et al., 2023). Over 1 billion hectares of land worldwide have been found to have primary salinization (Ivushkin et al., 2019). Out of 310 million hectares of irrigated land and 150 million hectares of dry agricultural land, 20% (62 million hectares) and 2% (32 million hectares) are respectively affected by secondary salinization (Qadir et al., 2014). It is expected that the continuous increase in the frequency and duration of droughts caused by global climate change will further exacerbate the increase in salinity in arid and coastal areas (Li et al., 2014; Daliakopoulos et al., 2016). The accumulation of soluble salts in the surface layer of the soil leads to poor physicochemical and structural characteristics of the soil, such as easy compaction, poor air and water permeability. It also tends to slow down the warming of the surface soil, reduce soil enzyme activity and soil fertility. Therefore, the increase in soil salinity has a serious negative impact on plant growth (Munns and Tester, 2008). As fixed organisms, plants must face various environmental challenges directly. Based on this background, to improve and utilize these marginal soils, it is extremely important to identify effective strategies for plant adaptation to salt stress and further develop potential methods to improve plant performance under salinity conditions.

Plants have evolved various survival mechanisms through long-term interactions with saline-alkali environments, including influencing soil microorganisms through host interaction (Bakker et al., 2018). Soil microbiome has been shown to have great potential in improving plant tolerance to abiotic stress and is expected to become a sustainable and effective strategy (Singh et al., 2023). Especially, a diverse group of root-related microorganisms is crucial for promoting plant adaptation to saline-alkali environments (Qin et al., 2016). Plant growth promoting rhizosphere bacteria (PGPR) have been recognized as important biological tools for alleviating salt stress in plants (Kumar et al., 2020; Girma et al., 2022). They can improve plant performance under salinity conditions through several beneficial processes, such as mediating ion homeostasis, producing plant hormones, promoting osmotic accumulation, enhancing antioxidant activity, and enhancing nutrient absorption (Paul and Lade, 2014; Gil et al., 2023). In addition, various fungi in plant roots, such as mycorrhizal and endophytic fungi, play a key role in improving plant salt adaptation, productivity, and abiotic stress tolerance (Dastogeer et al., 2020; Siddiqui et al., 2022). Therefore, the use of microbial communities to mediate plant tolerance is receiving increasing attention (Busby et al., 2017). So far, the physiological and molecular basis of plant adaptation to salt has been fully demonstrated (Zhu, 2016). Undoubtedly, salt-tolerant plants are more adaptable to salt stress than salt-sensitive plants. Previously, it was generally believed that this result was driven by genetic differentiation (Zhang et al., 2022), but recent studies have shown that the specific enrichment of rhizosphere microbiota driven by varieties may be a key factor determining their salt tolerance (Li H. et al., 2021; Wu et al., 2022). Indeed, soil type determines the overall structure and composition of rhizosphere microbial communities, while host genotype to some extent determines their specific species composition and abundance (Bulgarelli et al., 2012). That is, root-associated microbial communities are shaped to some extent by host genetics, and there is a strong correlation between host genetic differences and microbiome composition (Schlaeppi et al., 2014; Naylor et al., 2017). Due to their genetic composition and subsequent differences in the composition and content of root exudates, stress-tolerant and sensitive varieties of plants may attract different microbial communities (Naylor et al., 2017; Bont et al., 2020), leading to varying degrees of stress resistance. Therefore, identifying relevant microorganisms and their recruitment pathways is crucial for understanding some of the mechanisms behind salt tolerance or sensitivity in these plants.

Alfalfa (*Medicago sativa* L.) is a significant material for utilizing saline-alkali soil and studying strategies mediated by the aforementioned microbiota. Alfalfa is the most important and widely planted legume forage in the world (Shen et al., 2020). Such as for soil and water conservation, plant restoration, and improving soil conditions (Fernandez et al., 2019; Saidi et al., 2021), etc. In China, alfalfa is usually planted in arid inland areas in the north, accounting for approximately 90% of the country's alfalfa planting area. However, with global climate change leading to a gradual increase in the degree of salinization in arid inland areas in the north, salinity and alkalinity can lead to a significant decrease in alfalfa yield and quality, and even inability to grow in certain severely saline areas. Alfalfa cultivation faces even more severe challenges. At present, a large number of studies have described the use of traditional breeding, as well as various genetic engineering and molecular breeding methods to improve alfalfa yield and cultivate varieties resistant to abiotic stress (Shi et al., 2017). However, compared to plants such as rice (Lian et al., 2020), tomato (Deng et al., 2021), wheat (Jochum et al., 2019), and maize (Carter et al., 2023), there is still a significant knowledge gap regarding the potential function of alfalfa rhizosphere microorganisms in improving their resistance.

Given that the microbiome has the potential to be the second genome of plants, it has been shown to have great potential in improving plant resistance, In the future, it is expected that plant roots can be genetically engineered to recruit beneficial microbiota to improve resistance and yield through regulating rhizosphere secretions (Mavrodi et al., 2021). Of course, there is still a lot of basic work to be done before achieving this goal. We have previously demonstrated that soil microbiome can significantly improve alfalfa drought adaptability, and this effect exhibits variety-specific differences (Fan et al., 2023). However, it is still unknown whether there is still variety specific recruitment of rhizosphere microorganisms to improve their salt tolerance under salinity stress. Deeply understanding the differences in rhizosphere microbial composition, abundance, and function among different alfalfa varieties on saline-alkali soil is an important step in applying it to agriculture. For this purpose, we selected alfalfa varieties with different salt tolerance, including the strong salt- tolerant variety "Barricade (BB)", the medium salt-tolerant variety "Zhongmu No.1" (ZM), and the salt-sensitive variety "Aohan (AH)", and studied their growth, yield, and rhizosphere microbial community under different degrees of salt alkali conditions. We focus on exploring: (1) What changes occur in alfalfa growth, yield, and rhizosphere microbial structure with the increase of salinity?, (2) Did different salt-tolerant alfalfa varieties shape different rhizosphere microbial communities in response to salinity stress?, (3) Identify the dominant microbial populations of salt- tolerant plant species and how they can improve alfalfa salt tolerance? We aim to reveal the regulatory mechanism of plant microbial interactions in alfalfa response to salt stress mediated by varieties.

## Materials and methods

### Experimental area, variety selection, and experimental design

The experimental area is located in Ganzhaomiao Town (107 ° 25 ′ 3113 ″ E, 40 ° 82 ′ 7061 ″ N), Linhe District, Bayannur City, in the Inner Mongolia Autonomous Region of China. The area of saline-alkali land in this area is about 300 hm^2^, and a representative 2000 m^2^ was selected as the experimental area. The soil type is saline clay. According to the total salt content of the soil, the experimental site was divided into mild (1 g/kg ≤ total salt ≤ 3 g/kg, 7.1 ≤ pH ≤ 8.5, BM_Mi), moderate (3 g/kg<total salt ≤ 6 g/kg, 8.5<pH ≤ 9.5, BM_Mo), and severe (total salt>6 g/kg, pH>9.5, BM_Se) saline-alkali land.

Based on a large number of field experiments in the early stage, strong salt-tolerant variety (*Medicago sativa* L. 'Barricade', BB), medium salt-tolerant variety Zhongmu No.1 (*Medicago sativa* L. ‘Zhongmu No.1’, ZM), and salt-sensitive variety Aohan (*Medicago sativa* L. ‘Aohan’, AH) were selected. The seeds are all sourced from the Grassland Research Institute of the Chinese Academy of Agricultural Sciences. The salt-tolerant variety Zhongmu No.3 (*Medicago sativa* L. ‘Zhongmu No.3’, ZM3) (Ma et al., 2017) was used as the control (CK), and the seeds were sourced from the Institute of Animal Husbandry, Chinese Academy of Agricultural Sciences. Before the experiment, variety identification was conducted on the seeds, and the purity and germination rate of the seeds met the sowing quality standards. In the experimental area, we divided the different degrees of salinity into plots, plot area 6 m×9 m, plot to plot spacing of 60 cm. Alfalfa was planted in 2020. Randomly plant 3 plots for each salinity level and variety.

### Determination of alfalfa growth indicators and collection of rhizosphere soil samples

Three points were selected in each plot and the number of emergence and preservation plants (calculated based on the number of main stems) of each variety were accurately recorded using the labeling method, and the emergence rate and preservation rate were calculated. The yield was measured during the initial flowering period (when the flowering ratio was less than 10%) of 2021 and 2022. Specifically, we selected 1 m×1 m quadrat in each plot and measured fresh grass yield after cutting, and then took 1 kg of fresh grass and placed it in an oven, after drying at 80° C for 16 hours, we measured its dry weight. The rhizosphere soil samples were collected during the early flowering period of alfalfa growth in 2022. We collected soil samples from the soil attached to the surface of alfalfa roots, and the soil closely attached to the roots is considered rhizosphere soil. We selected 5 plants with uniform growth from each variety in each degree of saline-alkali land, dug the roots with a sterile shovel, shook off the loose soil, and collected the rhizosphere soil attached to the root surface with a sterile brush. Finally, a total of 36 soil samples (4 replicates per group) were collected from different varieties of saline-alkali soil of different degrees (Mi_BB, Mi_ZM, Mi_AH, Mo_BB, Mo_ZM, Mo_AH, Se_AH, Se_ZM, Se_AH). Each soil sample was divided into two sub-samples. One of them was placed in a 5mL sterile frozen tube, frozen in liquid nitrogen, immediately sent back to the laboratory on dry ice, and stored at -80°C for the extraction of rhizosphere microbial DNA, which was completed within 24 hours (Zhao et al., 2020). The other part was placed in a plastic bag and transported to the laboratory at 4° C for storage at -20° C for subsequent measurement of soil physical and chemical properties as well as enzyme activity.

### Analysis of soil physicochemical properties and enzyme activity

The physicochemical properties of the soil were analyzed and tested by the Analysis and Testing Center of the Institute of Grassland Research, Chinese Academy of Agricultural Sciences (Hohhot, China). Specifically, the soil moisture content (MC) was determined by placing 5 g in an oven and drying it to a constant weight at 105°C ± 1°C, followed by weighing. The soil pH and electrical conductivity (EC) were determined in a mixture with a soil/water ratio of 1:5 (wt/vol) using a pH meter (Thermo Orion Star A111; Thermo Fisher, Germany) and an EC meter (ST3100C/F; OHAUS, U.S.). The soil total salt content (TS) was determined by the residue drying method. The soil total carbon (TC) and total nitrogen (TN) concentrations were measured using a CHNOS elemental analyzer (Vario MACRO cube, Elementar, Germany). Soil total phosphorus (TP) was assayed using a continuous flow analytical system (AA3, SEAL, Germany). Soil total potassium (TK) was quantified using inductively coupled plasma-atomic emission spectrometry (ICPS-7500, Shimadzu, Japan). Soil available nitrogen (AN) was detected using an alkaline hydrolysis diffusion method. Soil organic carbon (OC) was determined by the potassium dichromate sulfuric acid oxidation method. The available phosphorus (AP) content of the soil samples was determined by the molybdenum–antimony anti-colorimetric method. The soil available potassium (AK) content was determined by the ammonium acetate flame photometer method. Three biological replicates per sample were tested.

Enzyme activities were determined using the Soil Enzyme Activity Assay Kits (Solarbio, China). The activities of soil β-glucosidase (S-β-GC) (Catalog Number: BC0165), soil β-xylosidase (S-β-XYS) (Catalog Number: BC4015), soil cellulase (S-CL) (Catalog Number: BC0155), soil urease (S-UE) (Catalog Number: BC0125), soil alkaline protease (S-ALPT) (Catalog Number: BC0885), soil dehydrogenase (S-DHA) (Catalog Number: BC0395), and soil alkaline phosphatase (S-ALP) (Catalog Number: BC0285) were measured according to the instructions provided in the assay kit manual.

### DNA extraction, 16S and ITS amplification, sequencing, and data processing

Microbial community total genomic DNA extraction was performed according to the protocol provided by the DNeasy® PowerSoil® Pro Kit (QIAGEN, U.S.). The quality of the extracted genomic DNA was assessed by 1% agarose gel electrophoresis, and DNA concentration and purity were determined using a NanoDrop 2000 spectrophotometer (Thermo Scientific, U.S.). The V3-V4 variable region of the bacterial 16S rRNA gene and the fungal ITS rRNA gene ITS region were separately amplified using the ABI GeneAmp® 9700 PCR system (ABI, CA, U.S.) with the following primers: 338F (5’-ACTCCTACGGGAGGCAGCAG-3’)/806R (5’-GGACTACHVGGGTWTCTAAT-3’) for bacteria and ITS1F (5'-CTTGGTCATTTAGAGGAAGTAA-3')/ITS2R (5'-GCTGCGTTCTTCATCGATGC-3') for fungi (Wang et al., 2022). The PCR reaction system (20 µL) consisted of 1× FastPfu buffer, 250 mM deoxynucleoside triphosphates (dNTPs), 0.2 mM of each primer, 1 U of FastPfu polymerase, and 10 ng of template DNA. PCR was carried out in triplicate with the following thermal cycling conditions: an initial denaturation at 95°C for 3 min, followed by 27 cycles of denaturation at 95°C for 30 s, annealing at 55°C for 30 s, extension at 72°C for 45 s, and a final extension step at 72°C for 10 min. Subsequently, PCR products were purified, pooled with the equimolar concentrations, and sequenced on the Illumina MiSeq PE300 platform at the Majorbio Bio-Pharm Technology Co., Ltd. (Shanghai, China).

Raw sequencing reads were subjected to quality control using fastp (Version 0.19.6^1^) (Chen et al., 2018), Reads containing N or quality score < 20 or with a length < 50 bp were discarded. Subsequently, the paired-end sequences were merged to a single sequence with length of ~300 bp using FLASH (Version 1.2.11^2^) (Magoč and Salzberg, 2011). Then the optimized sequences were clustered into operational taxonomic units (OTUs) using UPARSE 7.1 with 97% sequence similarity level (Edgar, 2013). To minimize the impact of sequencing depth on the measurements of alpha and beta diversity, 16S rRNA gene sequences from each sample were rarefied to the minimum sequence count per sample. Representative sequences were classified using RDP Classifier (Version 2.2^3^) (Wang et al., 2007) and annotated against the SILVA reference database (v138) with a confidence threshold of 0.7. Singleton OTUs and OTUs being annotated as plant mitochondria or chloroplast were discarded.

### Statistical and bioinformatics analyses

Statistical analysis was carried out using SPSS software (v.26.0.0.2). Analysis of variance (ANOVA) was employed to assess the significance of differences among various groups (*p<0.05, **p<0.01, and ***p<0.001 were considered to be significant). Bar graphs were plotted with Prism 9.5 (GraphPad Software, LLC). Alpha diversity for each sample was assessed based on four diversity indices: Chao1, Shannon, Simpson, and Ace. All sample indexes were calculated using QIIME (v.1.9.1) (Caporaso et al., 2010), and Kruskal-Wallis H test was used to evaluate the differences in alpha diversity index between different treatments. Rarefaction curves based on the Shannon and Ace indices were plotted for each sample, assessing the effectiveness of sequencing depth. Principal Coordinate Analysis (PCoA) based on the Bray-Curtis distance was performed using the R Package "vegan (v2.5-3)" to assess the similarity of microbial community structures among samples (Oksanen et al., 2016). Additionally, we performed a non-parametric PERMANOVA analysis to determine the significance of differences in microbial community structures between sample groups. Species composition circle plots, distribution histograms, heatmaps, and correlation heatmaps between species and environmental factors were generated with the ggplot2 (v.3.3.5) package. The correlations between soil factors and among soil factors and the root-associated microbial communities of the three alfalfa varieties were calculated by the "linkET" package in R, and generated a mantel test network graph. Visualize the shared and unique microbial taxa among different treatment samples based on a Venn diagram. Identify significantly different species between groups using the ANCOM differential analysis provided by QIIME2. Linear discriminant analysis effect size (Lefse), for which the logarithmic LDA score was set to 3.0 with statistical significance (*p* < 0.05), and the functional potential of a bacterial community predicted by PICRUSt2 were both performed on the Majorbio Cloud platform^4^.

## Results

### Differences in growth and production performance of different salt tolerant varieties in degrees of saline-alkali soil

**Figure 1:**
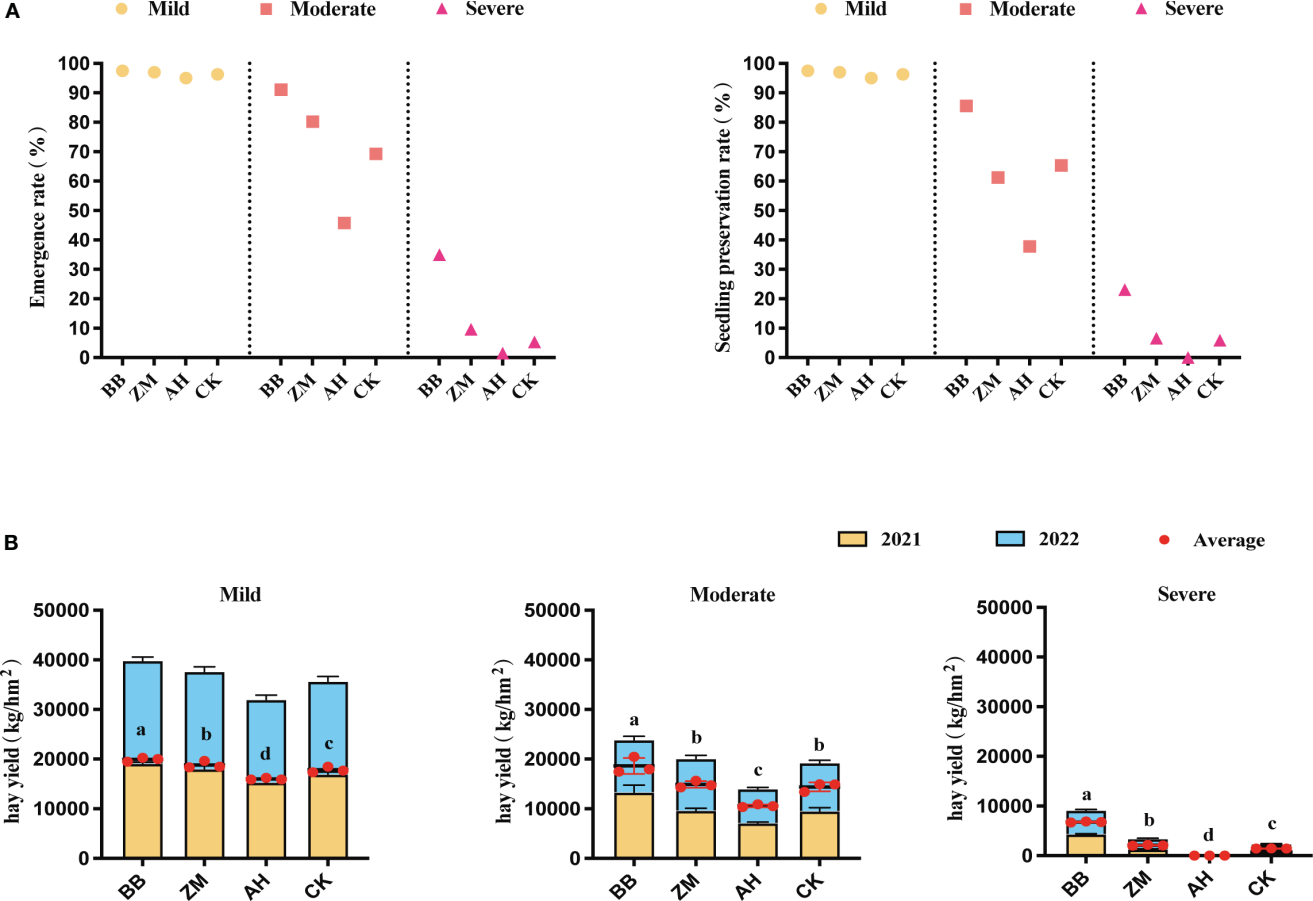
Seedling emergence rate, seedling preservation rate, and yield. **(A)** The emergence rate and seedling preservation rate of alfalfa with different salt tolerance on different degrees of saline-alkali soil, **(B)** Hay yield of alfalfa with different salt tolerance on different degrees of saline-alkali soil. ANOVA analysis was used to analysis the effects of salinity stress and variety on seedling emergence rate, seedling preservation rate, and yield. Error bars represent SD values of three replicates. Different letters indicate significant differences (*p*<0.05, Duncan’s multiple range test).

The emergence rate and seedling preservation rate of different salt-tolerant varieties were significantly different in different degrees of saline-alkali soil (**Figure 1A**). Under mild saline-alkali conditions, there was little difference among different varieties; Under moderate saline-alkali conditions, the emergence and preservation rates of various varieties were affected to varying degrees, with BB had a minimum impact of 91.1% and 85.6% respectively, followed by ZM, both higher than CK, while AH having a significant impact, significantly lower than CK; Under severe saline-alkali conditions, the emergence rate and seedling preservation rate of various varieties were severely inhibited, especially for AH, which had an emergence rate of only 1.5% and a seedling preservation rate of 0%. However, the emergence rate and seedling preservation rate of BB were 35% and 23.1%, respectively, which were relatively better than CK and the other two varieties.

Salinity seriously affected the yield of alfalfa, and different salt-tolerant varieties had significant differences in hay yield on different degrees of saline-alkali soil (**Figure 1B**). The average two-year hay yield results showed that BB maintained a relatively high yield in various degrees of saline-alkali soil, significantly higher than CK and other varieties; The hay yield of AH was the lowest, significantly lower than CK and other varieties (*p*<0.05). Especially in severe saline-alkali soil, the hay yield of BB was 6784.33 kg/hm^2^, which was 3.25 times that of ZM, while AH cannot survive in severe saline-alkali soil.

### Differences in soil physicochemical properties and enzyme activity in saline-alkali soils of different degrees

**Figure 2:**
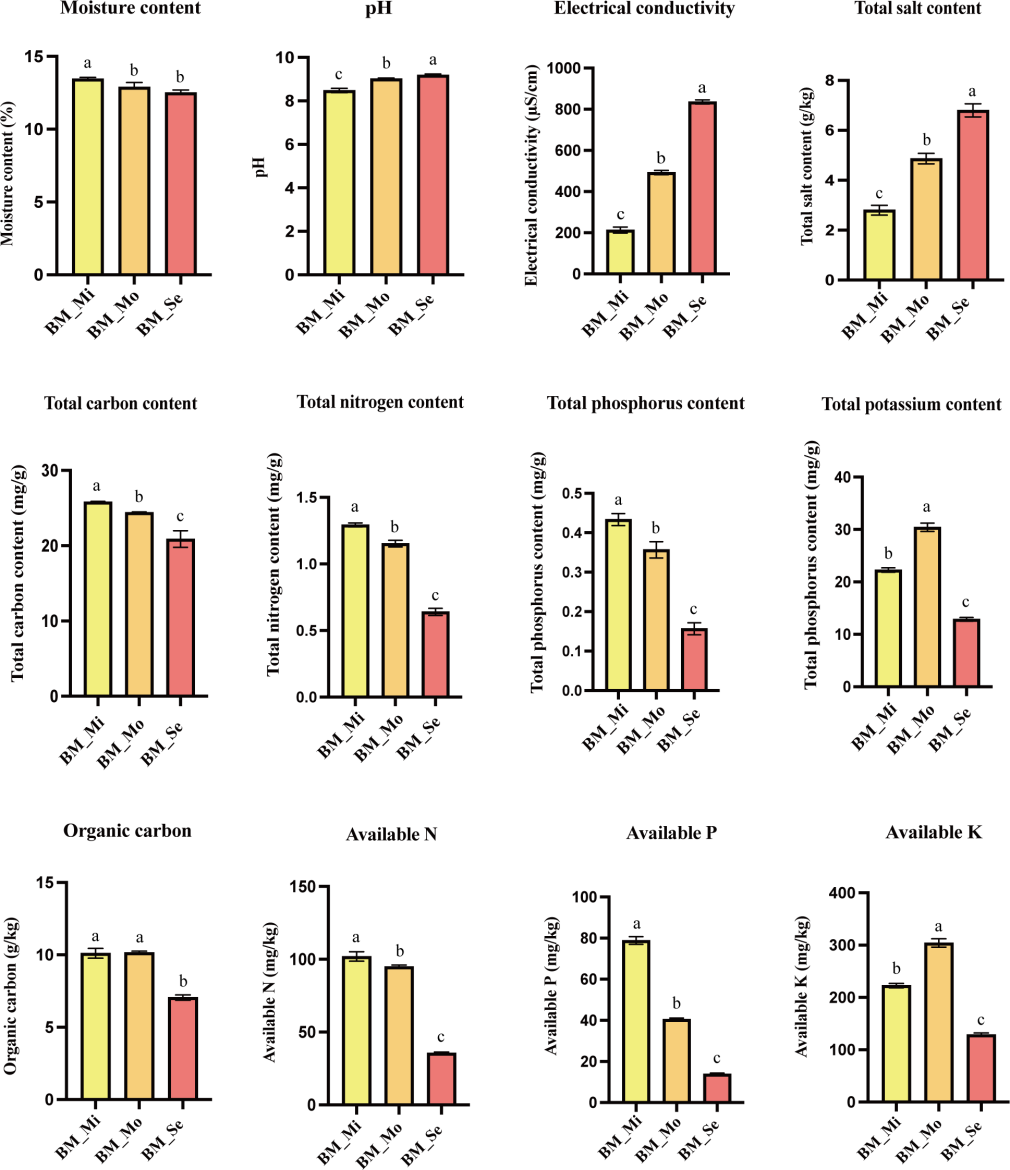
Soil physicochemical properties of saline-alkaline land with different degrees. ANOVA was used to analyse the differences in soil physical and chemical properties of saline-alkali soils with different degrees. Error bars represent SD values of three replicates. Different letters indicate significant differences (*p*<0.05, Duncan’s multiple range test).

The physicochemical properties of soils from different degrees of saline-alkali land exhibited significant variations (**Figure 2**). MC in BM_Mi was significantly higher at 13.45% in comparison to BM_Mo (12.9%) and BM_Se (12.5%) (*p* < 0.05). pH, EL, and TS showed a consistent trend, which significantly increased with the increase in salinity. Specifically, the pH and TS of BM_Mi, BM_Mo, and BM_Se were 8.48, 2.8 g/kg, 9.02, 4.87 g/kg, and 6.8 g/kg, 9.19, respectively, with statistically significant inter-group differences (*p* < 0.05). Moreover, the content of TN, TP, AN, and AP all showed a gradient decreasing trend with the increase of salinity, and there was a significant difference between groups (*p* <0.05). Likewise, TC and OC contents also showed a decreasing trend, with BM_Se having significantly lower TC and OC contents compared to BM_Mi and BM_Mo (*p* < 0.05). Furthermore, the TK and AK content were highest in BM_Mo at 30.4 mg/g and 304 mg/kg, followed by BM_Mi, with BM_Se having the lowest values, and significant differences were observed among the groups (*p* <0.05).

**Figure 3:**
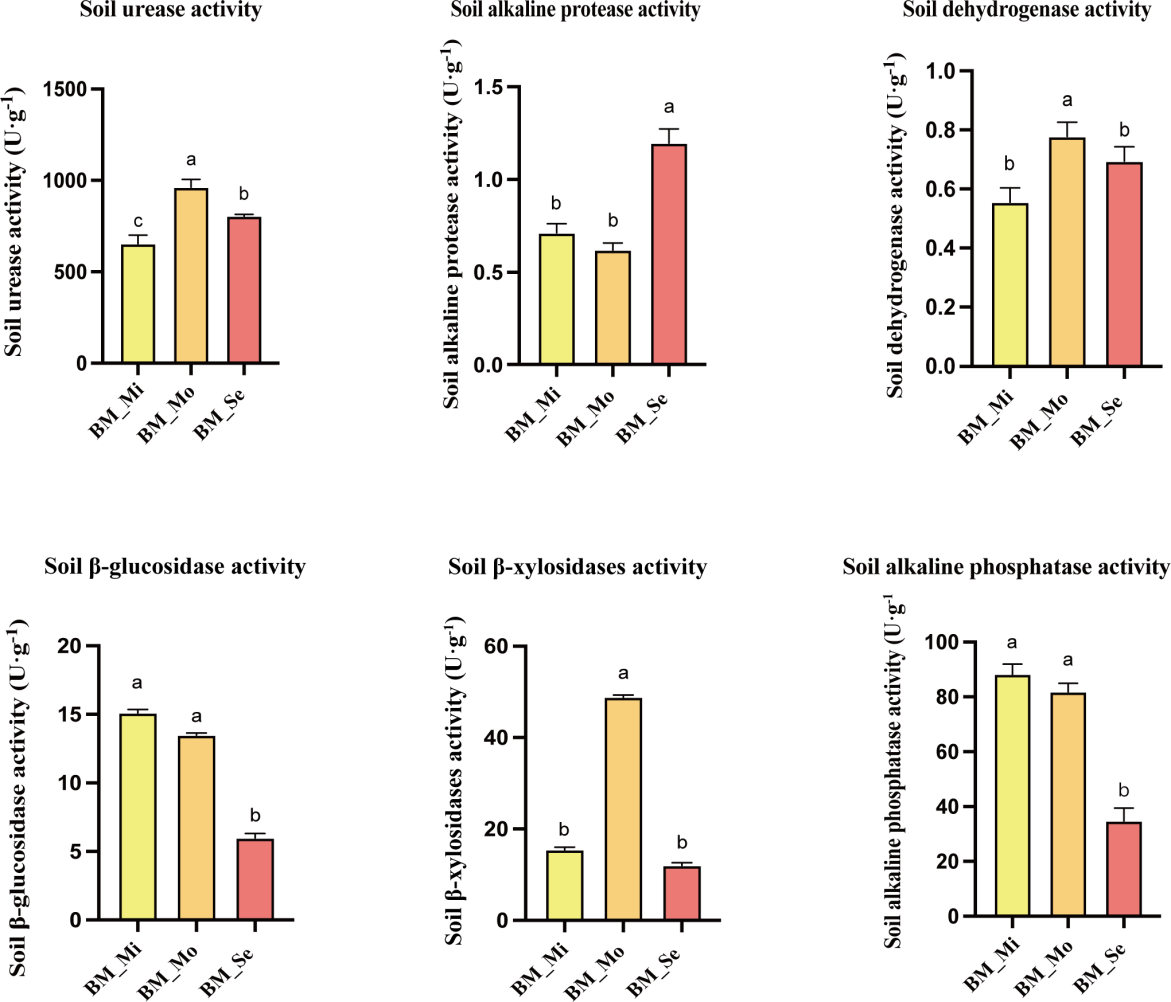
Soil enzyme activity in saline-alkali soil of different degrees. ANOVA was used to analyze the differences in soil enzyme activity of saline-alkali soils with different degrees. Error bars represent SD values of three replicates. Different letters indicate significant differences (*p*<0.05, Duncan’s multiple range test).

Differences in soil enzyme activities among various degrees of saline-alkali soils were also observed (**Figure 3**). The enzymatic activities of S-DHA (0.78 U·g^-1^) and S-β-XYS (48.81 U·g^-1^) in BM_Mo were significantly higher than those in BM_Mi and BM_Se (P < 0.05), while no significant difference was observed between BM_Mi and BM_Se (*p* > 0.05). BM_ Mo had the highest S-UE activity at 963.35 U·g^-1^, followed by BM_ Se (805.44 U·g^-1^), BM_ Mi (653.9 U·g-1) was the lowest, with significant differences between groups (*p* <0.05). Meanwhile, BM_Se showed significantly lower S-β-GC (5.98 U·g-1) and S-ALP (34.77 U·g-1) activities compared to BM_Mi and BM_Mo, with no significant difference observed between BM_Mi and BM_Mo (*p* > 0.05). In BM_Se, only the S-ALPT activity (1.2 U·g-1) was significantly higher than in BM_Mi (0.71 U·g-1) and BM_Mo (0.62 U·g-1) (*p* <0.05).

### Microbial community alpha-diversity and variation of community composition

High-quality bacterial sequences totaling 1,425,284 reads and fungal sequences totaling 1,646,829 reads were obtained from all soil samples using the Illumina MiSeq platform. All samples showed stable rarefaction curves (**Supplementary Figure 2**), indicating sufficient sequencing depth. To ensure the validity and accuracy of the data analysis, all samples were rarefied to the minimum sequence count. For bacterial and fungal analyses, 22,331 and 37,059 high-quality sequences were retained for further analysis, respectively.

**Figure 4:**
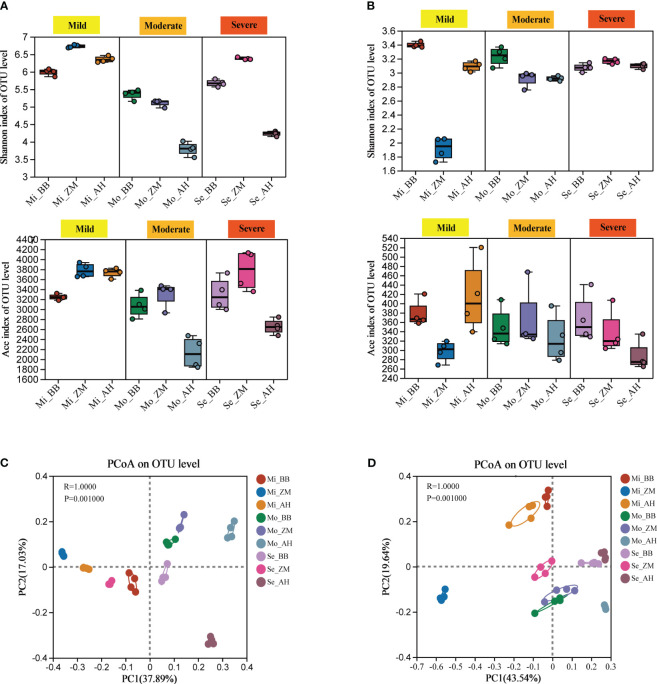
Microbial diversity and PCoA analysis of different varieties in different degrees of saline-alkali soil. **(A)** Changes in bacterial chao1 and Ace index at the out level, **(B)** Changes in fungal chao1 and Ace index at the out level, **(C)** PCoA of bacterial community in all samples based on Bray-Curtis distance, **(D)** PCoA of fungal community in all samples based on Bray-Curtis distance.

We observed significant variations in the α-diversity indices of root-associated bacterial and fungal communities among different salt-tolerant alfalfa varieties grown in saline-alkali soils with different degrees (**Supplementary Table S1**). The Shannon and Ace indices were employed to illustrate the diversity and richness of microbial communities in the alfalfa rhizosphere under different treatments. Inter-group differences based on the Shannon and Ace indices were assessed using the Kruskal-Wallis test. For bacterial communities, in mildly saline-alkali soils, BB showed significantly lower diversity compared to the other two varieties. In moderately and severely saline-alkali soils, both BB and ZM showed significantly higher diversity than AH (*p* < 0.05). Notably, AH showed a substantial decrease in bacterial diversity in moderately and severely saline-alkali soils (**Figure 4A**, **Supplementary Figure 3A**). In the case of fungal communities, the Shannon index showed significant differences among the various alfalfa varieties in both mild and moderate saline-alkali soils, with BB significantly higher than AH and ZM (*p* < 0.05). In severe saline-alkali soil, ZM exhibited significantly higher Shannon index values than the other two varieties (*p* < 0.05). The Ace index, on the other hand, was only significantly higher in AH compared to ZM in mild saline-alkali soil (*p* < 0.05), with no significant differences among varieties observed in severe and moderate saline-alkali soils (*p* > 0.05) (**Figure 4B**, **Supplementary Figure 3B**).

Based on Bray-Curtis distance combined with ANOSIM analysis, PCoA analysis revealed significant differences in community structure among different varieties in saline-alkali soils of varying degrees. The variation explained by PC1 in the bacterial community was 37.89%, while PC2 explained 17.03% of the total variation. Each group was distinctly separated along PC1, indicating differences in rhizosphere microbial composition among different varieties in saline-alkali soils of varying degrees (**Figure 4C**). Specifically, the distance between AH with different degrees of salinity was relatively far, indicating the greatest difference in species composition, and also indicated that they were more sensitive to salinity and are greatly affected by it. Next was ZM, and the distance between the three groups was closer than AH. The distance between the three groups of BB with different degrees of salinity was relatively close, indicating that their species composition was relatively stable and they were more tolerant to salinity; The variation explained by PC1 in the fungal community was 43.54%, and PC2 explained 19.64% of the total variation. In mild saline-alkali soil, ZM was far away from BB and AH, while in moderate and severe saline-alkali soil, the distance between the three varieties was relatively close, especially in moderate saline-alkali soil, where BB and ZM species composition are relatively similar (**Figure 4D**).

**Figure 5:**
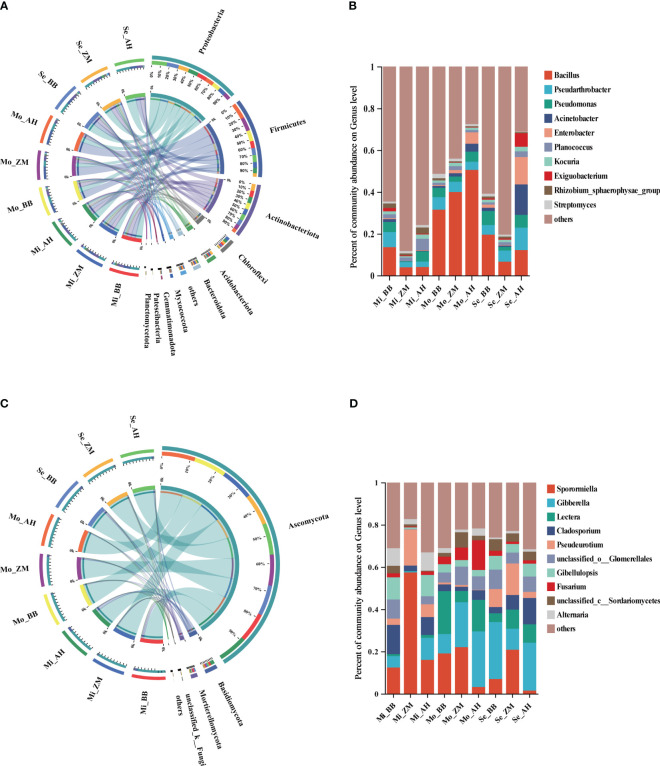
The composition of rhizosphere microbial communities varies among different varieties in different degrees of saline-alkali soil. **(A)** The bacterial community composition (relative abundance top 10) at the phylum level, **(B)** The bacterial community composition (relative abundance top 10) at the genus level, **(C)** The fungal community composition (relative abundance top 10) at the phylum level, **(D)** The fungal community composition (relative abundance top 10) at the genus level.

The rhizosphere bacteria composition of three different alfalfa varieties with varying salt tolerance showed notable differences in soils with varying degrees of salinity. The top three phyla with relatively higher abundances were *Proteobacteria*, *Firmicutes*, and *Actinobacteriota* (**Figure 5A**). We observed a significant increase in the relative abundance of *Firmicutes* in the rhizosphere of alfalfa in moderately saline-alkaline soils, whereas *Proteobacteria* showed a pronounced increase in severe saline-alkaline soils (**Supplementary Figure 4A**). In mildly saline-alkaline soils, there were minimal differences among the three varieties. In moderately saline-alkaline soils, both ZM and AH showed a substantial increase in Firmicutes, with a corresponding decrease in *Actinobacteriota* in AH. In severe saline-alkaline soils, the relative abundance of *Firmicutes* decreased across all three varieties, while *Proteobacteria* significantly increased in AH. However, overall bacterial diversity notably decreased in highly saline-alkaline soils.

The top three genera with relatively higher abundances were *Bacillus*, *Pseudarthrobacter*, and *Pseudomonas* (**Figure 5B**). Notably, in mildly saline-alkaline soils, BB showed significantly higher relative abundances of *Bacillus* and *Pseudarthrobacter* compared to ZM and AH. In moderately saline-alkaline soils, the abundance of *Bacillus* increased significantly in all three varieties, with AH > ZM > BB, and AH showed an increase in the abundance of *Enterobacter*. In severe saline-alkaline soils, the relative abundance of *Bacillus* decreased significantly, but BB's relative abundance of *Bacillus* remained notably higher than that of ZM and AH, whereas AH showed higher relative abundances of *Enterobacter* and *Acinetobacter*. Furthermore, we observed that the relative abundance of *Pseudomonas* in the rhizosphere of BB and AH was significantly higher than that in ZM, and this relative abundance remained relatively stable. Additionally, a heatmap of the top 30 bacterial genera based on abundance was generated to further investigate the composition of rhizosphere bacteria in alfalfa (**Supplementary Figure 5A**). It was evident that there were differences in the abundance of rhizosphere bacteria among the three varieties across soils with varying degrees of salinity. Most genera in Mo_AH and Se_AH exhibited lower relative abundances, indicating that moderate saline-alkaline conditions already had a significant impact on these varieties. On the other hand, Se_BB and Se_ZM showed higher abundances of various genera, suggesting that BB and ZM have stronger saline-alkaline tolerance than AH. Additionally, in severe saline-alkaline soils, BB and ZM exhibited higher abundances of genera such as *Ensifer*, *Azoarcus*, *Streptomyces*, *Novosphingobium*, and *Sphingomonas* compared to AH.

We further analyzed the rhizosphere fungal composition of three alfalfa varieties with varying salt tolerance across soils with different degrees of salinity. The top three phyla with relatively higher abundances were *Ascomycota*, *Basidiomycota*, and *Mortierellomycota* (**Figure 5C**). It was evident that *Ascomycota* dominated and exhibited higher relative abundances in the rhizospheres of all varieties across different saline-alkaline levels, establishing an absolute dominance, with only a slight enrichment of *Basidiomycota* in the rhizosphere of AH (**Supplementary Figure 4B**). The top three genera with relatively higher abundances were *Sporormiella*, *Gibberella*, and *Lectera* (**Figure 5D**). In mildly saline-alkaline soils, ZM showed significantly higher relative abundances of *Sporormiella* and *Pseudeurotium* compared to BB and AH. In moderately saline-alkaline soils, AH exhibited a marked decrease in the relative abundance of *Sporormiella*, while *Gibberella*, *Lecter*a, and *Fusarium* significantly increased. In severe saline-alkaline soils, BB had a higher abundance of *Gibberella*, ZM had a higher abundance of *Sporormiella*, and we observed an increase in the relative abundance of *Cladosporium* in AH and ZM. To provide a comprehensive understanding of the rhizosphere fungal composition, a heatmap of the top 30 fungal genera based on abundance was generated (**Supplementary Figure 5B**). It was worth noting that while there were differences among the three varieties in mildly saline-alkaline soils, the overall differences among the three varieties in moderately and severe saline-alkaline soils were not substantial. Additionally, with the increasing saline-alkaline levels, AH's rhizosphere exhibited a greater enrichment of molds and potential pathogenic fungi.

### Relationships between microbial communities of different salt-tolerant varieties and soil properties

**Figure 6:**
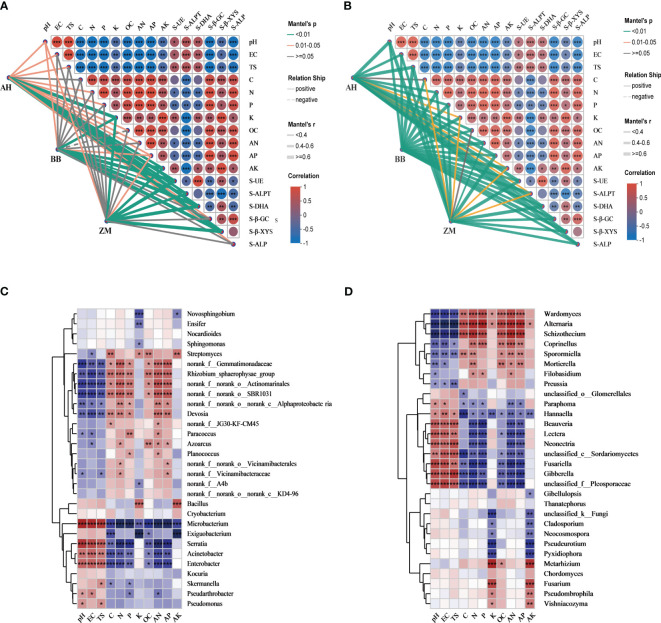
Interactions among soil physicochemical factors and their associations with soil enzyme activity and the rhizosphere microbial communities of different salt-tolerant varieties. **(A)** Mantel test revealed correlations between rhizosphere bacteria of different salt-tolerant varieties and the physicochemical properties of saline-alkaline soils, **(B)** Mantel test revealed correlations between rhizosphere fungi of different salt-tolerant varieties and the physicochemical properties of saline-alkaline soils, **(C)** Spearman correlation analyses were performed between the top 30 abundant bacterial genera and the physicochemical factors, **(D)** Spearman correlation analyses were performed between the top 30 abundant fungal genera and the physicochemical factors. The red and blue colors indicate positive and negative correlations, respectively. Different significance levels of correlation analyses are marked with asterisks (*, *p*<0.05; **, *p* < 0.01; ***, *p* < 0.001).

The Mantel test network (**Figures 6A, B**) and correlation heatmap (**Figures 6C, D**) were employed to assess the relationship between soil physicochemical properties and the rhizosphere microbial community structure of different salt-tolerant varieties. We initially observed a strong correlation among soil physicochemical properties, as well as with rhizosphere soil enzyme activities. pH, EC, and TS showed significant negative correlations with various soil nutrient indices. Additionally, they were positively correlated with carbon cycling (S-β-GC, S-β-XYS) and phosphorus cycling-related enzyme activity (S-ALP), while exhibiting significant positive correlations with nitrogen cycling-related enzyme activity (S-UE, S-ALPT, S-DHA) (**Figures 6A, B**). The relationship between rhizosphere bacterial communities and soil physicochemical properties varied among salt-tolerant varieties (**Figure 6A**). Although all three varieties were significantly influenced by pH, the salt-tolerant BB and ZM had no significant correlations with TS and EC, suggesting that they are less affected by salinity, whereas the salt-sensitive AH showed the opposite trend, indicating a greater impact of salinity. Furthermore, BB and ZM exhibited no significant correlations with C, N, P, OC, or AN, while AH had significant correlations with all nutrients except C. We also noted that K, AK, and AP had significant positive correlations with the rhizosphere of all three varieties. From the correlation heatmap, it can be observed that among the bacteria with relatively high abundance, most bacteria positively correlated with nutrient content were negatively correlated with pH, EC, and TS (**Figure 6C**). *Microbacterium*, *Serratia*, *Acinetobacter*, and *Enterobacter* showed highly significant positive correlations with pH, EC, and TS. *Bacillus* not only exhibited a certain positive correlation with pH, EC, and TS but also displayed highly significant positive correlations with K and AK content.

The relationship between rhizosphere fungal communities and soil physicochemical properties showed minimal differences among salt-tolerant varieties (**Figure 6B**). Rhizosphere fungal communities of all varieties exhibited significant correlations with soil physicochemical properties (except C and OC), indicating that changes in fungal communities were primarily induced by soil physicochemical properties, with a relatively weaker dominant effect of varieties. Among the fungi with relatively high abundance, eight, including *Beauveria*, *Neonetria*, and *Gibberella*, showed significant positive correlations with pH, EC, and TS, and significant negative correlations with C, N, P, AN, and AP. In contrast, seven fungi, including *Wardomyces*, *Alternaria*, and *Schizothecium*, showed the opposite trend (**Figure 6D**).

### Identification of shared, unique, and dominant microbial taxa in the rhizosphere soil of different salt-tolerant varieties

**Figure 7:**
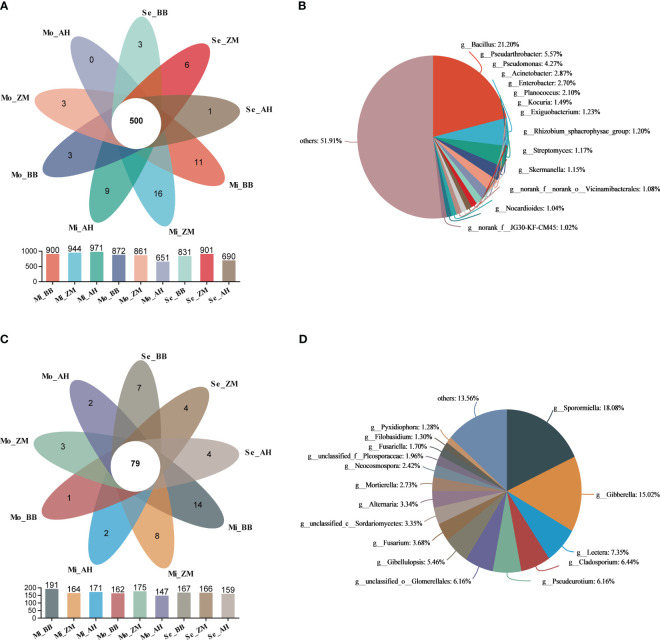
Analysis based on Venn to identify shared and unique microbial genera in the rhizosphere of different varieties in different degrees of saline-alkali soil. **(A)** Shared and unique bacteria in the rhizosphere of different salt-tolerant varieties in different degrees of saline-alkali soil, **(B)** The distribution and proportion of shared bacterial genera, **(C)** Shared and unique fungi in the rhizosphere of different salt-tolerant varieties in different degrees of saline-alkali soil, **(D)** The distribution and proportion of shared fungal genera.

The Venn diagram illustrates the shared and unique microbial information among the three varieties in soils with varying degrees of salinity. We observed that as salinity levels increased, the number of unique bacterial genera in the rhizosphere of different varieties decreased, with AH showing the most significant reduction (**Figure 7A**). Furthermore, we noted the presence of 500 genera that were consistently present in the rhizosphere of different varieties across soils with varying degrees of salinity. We defined these as the core microbiota of alfalfa rhizospheres in saline-alkaline soils. Among these 500 core bacterial genera, *Bacillus* (21.20%) was the most abundant, followed by *Pseudarthrobacter* (5.57%) and *Pseudomonas* (4.27%) (**Figure 7B**). On the other hand, there were more unique genera in the rhizosphere of different varieties in mildly saline-alkaline soils, and this number decreased notably in moderately and severe saline-alkaline soils. Across soils with varying degrees of salinity, different varieties shared 79 common fungal genera (**Figure 7C**). Among these, *Sporpmiella* (18.08%) and *Gibberella* (15.02%) were the predominant ones (**Figure 7D**).

We further conducted Venn analyses for different varieties across soils with varying degrees of salinity (**Supplementary Figure 6**). In mildly saline-alkaline soils, there were 772 bacterial genera shared among the three varieties, with BB, ZM, and AH having 43, 34, and 52 unique genera, respectively. Notably, ZM and AH had a higher number of shared genera compared to the other two varieties. In moderately and severe saline-alkaline soils, the number of shared bacterial genera among the three varieties decreased. Both BB and ZM exhibited an increase in the number of unique genera, while AH's unique genera significantly decreased to only 12. Additionally, BB and ZM had a higher number of shared genera, whereas the shared genera with AH were notably fewer (**Supplementary Figure 6A**). However, when it comes to fungi, the number of shared fungal genera among the three varieties in the rhizosphere remained relatively stable regardless of the salinity levels. BB exhibited a decrease in the number of unique fungal genera in moderately saline-alkaline soils, while AH showed an increase in the number of unique fungal genera in severe saline-alkaline soils.

**Figure 8:**
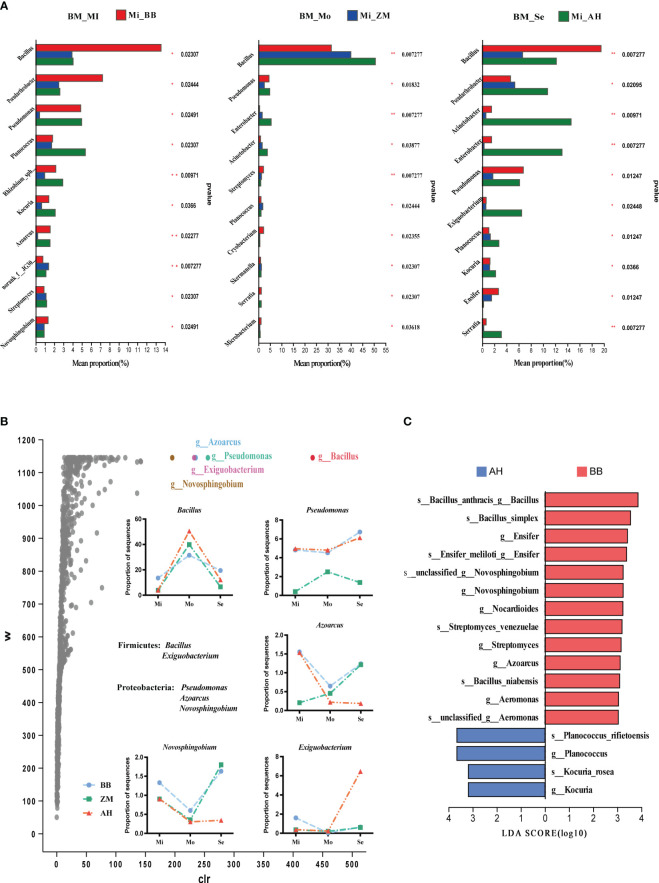
Differential analysis of rhizosphere bacteria in different salt-tolerant varieties under varying salinity conditions and identification of dominant bacteria in salt-tolerant and salt-sensitive varieties. **(A)** Bar chart illustrating inter-group differences in bacteria based on Kruskal-Wallis rank sum test. The Y-axis represents species names at the genus level, and the X-axis represents the average relative abundance within different groups. Different colors of bars indicate different groups; the far-right column represents the P-values, significantly enriched species in the rhizoplane soil are marked with asterisks. *p < 0.05, **p < 0.01. **(B)** Volcano plot of the ANCOM differential analysis of rhizosphere bacteria in different salt-tolerant varieties, along with the distribution of key differentially abundant genera in the rhizosphere of different salt-tolerant varieties across varying degrees of saline-alkaline soils. In the plot, points represent species, the Y-axis represents W values (a statistic measuring inter-group differences, with larger W values indicating greater differences between groups), and the X-axis represents clr values (center log transform), which indicate the degree of inter-group differences in sample abundance. Larger absolute values of clr indicate greater relative abundance differences. **(C)** Identification of characteristic dominant salt-tolerant bacterial genera for different salt-tolerant varieties using LEfSe analysis (LDA ≥ 3).

Inter-group differential analysis of rhizosphere microbiota of alfalfa varieties with different salt tolerance levels in various degrees of saline-alkaline soils (**Figure 8A**) was performed. We observed that *Bacillus* was the most abundant genus in the rhizosphere of alfalfa in saline-alkaline soils, significantly higher than other genera. Dominant bacteria varied significantly among different varieties. In mildly saline-alkaline soils, BB had significantly higher relative abundances of *Bacillus*, *Pseudarthrobacter*, and *Novosphingobium* compared to ZM and AH. Additionally, there were higher abundances of *Pseudomonas* and *Azoarcus* in BB, while ZM exhibited a significantly higher abundance of norank_f:*JG30-KF-CM45* than BB and AH. AH had significantly higher *Planococcus* compared to the other two varieties, and *Pseudomonas*, *Rhizobium_sphaerophysae_group*, *Kocuria*, and *Azoarcus* were significantly higher in abundance in ZM. In moderately saline-alkaline soils, AH showed significantly higher *Bacillus*, *Enterobacter*, and *Acinetobacter* abundances compared to BB and ZM. In severe saline-alkaline soils, we found that BB had significantly higher *Bacillus* and *Ensifer* abundances than ZM and AH. *Pseudomonas* also had higher abundances in BB, while most other genera were significantly higher in AH. We further conducted ANCOM differential analysis to identify dominant species (**Figure 8B**). The results indicated that *Bacillus*, *Pseudomonas*, *Azoarcus*, *Exiguobacterium*, and *Novosphingobium* showed significant inter-group differences. Among them, salt-tolerant BB and ZM exhibited more similar changes, differing significantly from AH. Under severe saline-alkaline conditions, salt-tolerant BB enriched higher abundances of *Bacillus*, *Pseudomonas*, *Azoarcus*, and *Novosphingobium* compared to salt-sensitive AH. Linear discriminant analysis (LDA) effect size (LEfSe) further confirmed that the mentioned genera were enriched in the rhizosphere of BB, including multiple species of *Bacillus* (**Figure 8C**).

**Figure 9:**
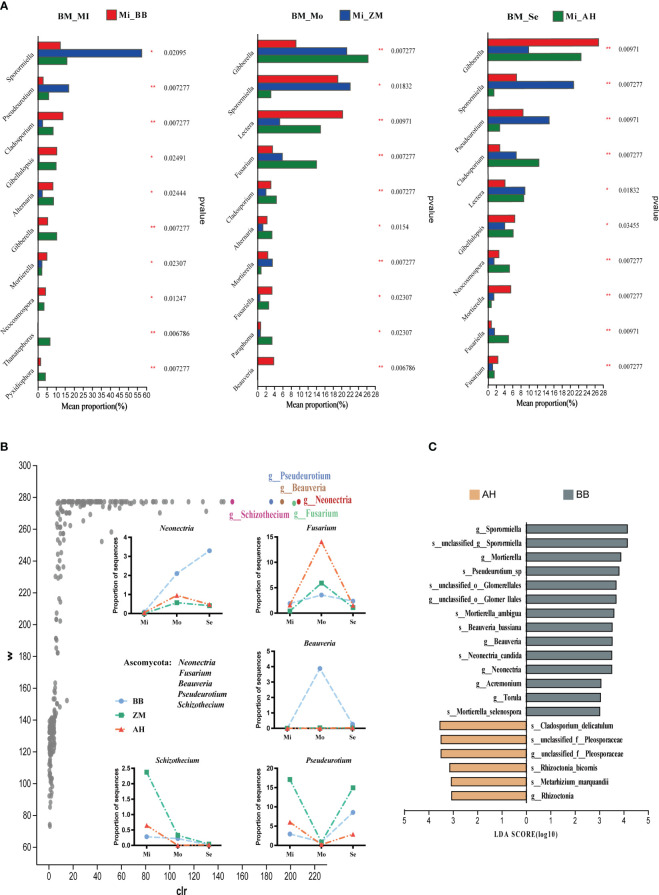
Differential analysis of rhizosphere fungi in different salt-tolerant varieties under varying salinity conditions and identification of dominant fungi in salt-tolerant and salt-sensitive varieties. **(A)** Bar chart illustrating inter-group differences in fungi based on Kruskal-Wallis rank sum test. The Y-axis represents species names at the genus level, and the X-axis represents the average relative abundance within different groups. Different colors of bars indicate different groups; the far-right column represents the P-values, significantly enriched species in the rhizoplane soil are marked with asterisks. *p < 0.05, **p < 0.01. **(B)** Volcano plot of the ANCOM differential analysis of rhizosphere fungi in different salt-tolerant varieties, along with the distribution of key differentially abundant genera in the rhizosphere of different salt-tolerant varieties across varying degrees of saline-alkaline soils. In the plot, points represent species, the Y-axis represents W values (a statistic measuring inter-group differences, with larger W values indicating greater differences between groups), and the X-axis represents clr values (center log transform), which indicate the degree of inter-group differences in sample abundance. Larger absolute values of clr indicate greater relative abundance differences. **(C)** Identification of characteristic dominant salt-tolerant fungal genera for different salt-tolerant varieties using LEfSe analysis (LDA ≥ 3).

Different salt-tolerant varieties also exhibited distinct dominant fungal communities (**Figure 9A**). *Sporormiella* and *Pseudeurotium* were dominant genera in the rhizosphere of ZM, with significantly higher abundances in mildly and severe saline-alkaline soils compared to BB and AH. *Gibberella* was the dominant genus in AH in mildly and moderately saline-alkaline soils, while becoming dominant in BB under severe saline-alkaline conditions. *Mortierella* was the dominant genus in the rhizosphere of BB in mildly and severely saline-alkaline soils, with higher abundances than AH in moderately saline-alkaline soils. Additionally, *Lectera* and *Beauveria* also exhibited higher abundances. *Cladosporium* and *Fusariella* were dominant genera in the rhizosphere of AH in moderately and severe saline-alkaline soils, with *Fusarium* significantly more abundant in AH than in BB and ZM in moderately saline-alkaline soils. ANCOM differential analysis results indicated that *Neonectria*, *Fusarium*, *Beauveria*, *Pseudeurotium*, and *Schizothecium* showed significant inter-group differences, and they exhibited similar trends with increasing salinity across the three varieties' rhizospheres, although with substantial differences in relative abundances (**Figure 9B**). Under moderately and severe saline-alkaline conditions, salt-tolerant BB enriched higher abundances of *Neonectria*, *Beauveria*, and *Pseudeurotium*, while AH enriched a substantial amount of *Fusarium*. Correspondingly, LEfSe further confirmed that *Sporormiella*, *Mortierella*, *Neonectria*, *Beauveria*, *Pseudeurotium* were enriched in the rhizosphere of salt-tolerant BB, while numerous pathogenic species, such as *Cladosporium_delicatulum* and *Rhizoctonia*, were significantly enriched in the rhizosphere of AH (**Figure 9C**).

### Function analysis of rhizosphere bacterial community in different alfalfa varieties in saline-alkaline soil

**Figure 10:**
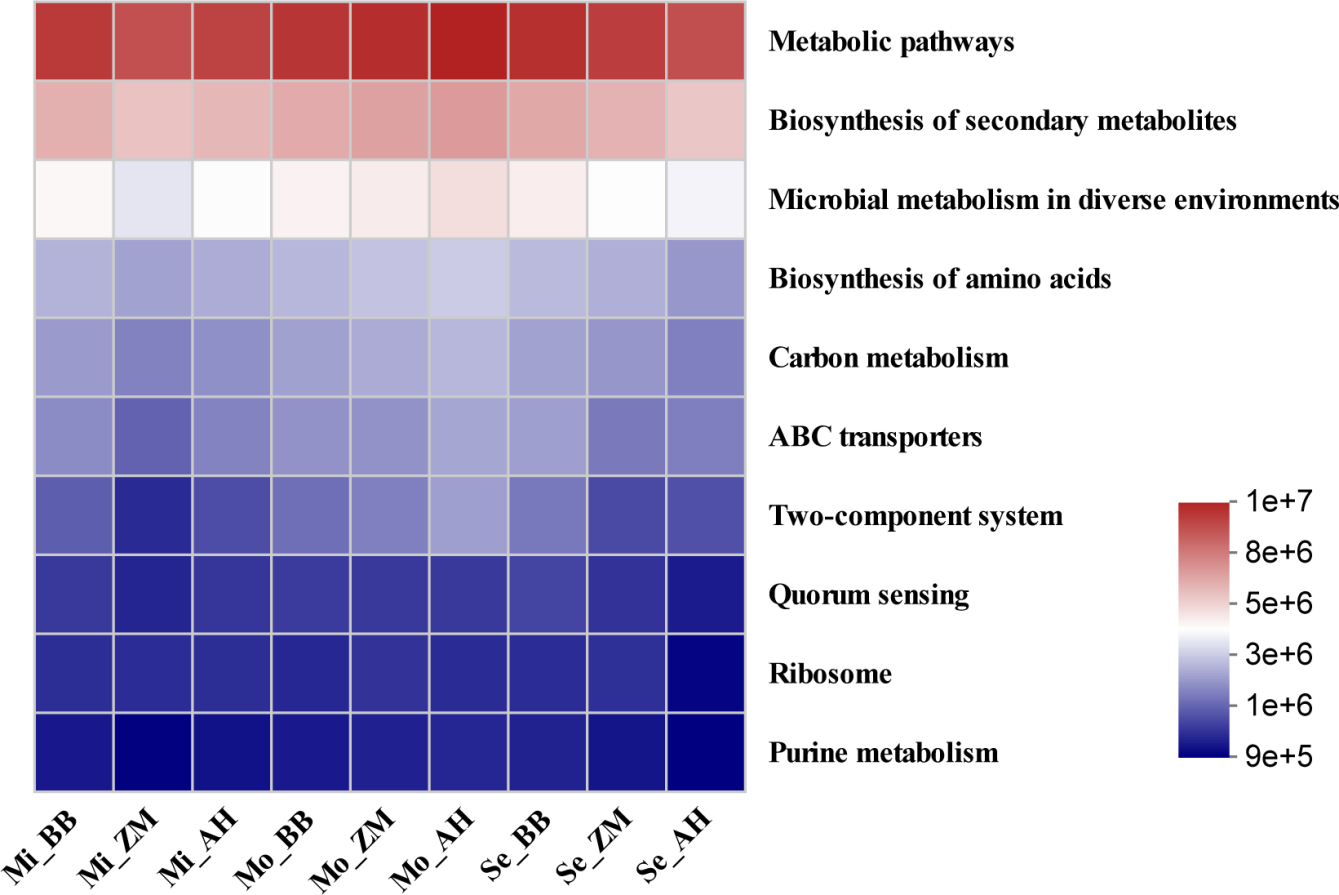
Function abundance heatmap of rhizosphere bacterial community in different alfalfa varieties in saline-alkaline soil.

As bacteria exhibit heightened sensitivity and play a more substantial role in plant adaptation to environmental stress, we conducted further statistical analysis of the functional abundance of KEGG pathways at level 3 within the bacterial communities of different alfalfa varieties in soils with varying degrees of salinity (**Figure 10**). We observed that metabolic pathways were the most abundant functional category in the rhizosphere bacterial communities of alfalfa. Additionally, functions related to the biosynthesis of secondary metabolites and microbial metabolism in diverse environments exhibited higher abundances. In mildly saline-alkaline soils, both BB and AH showed slightly superior functional profiles compared to ZM. In moderately saline-alkaline soils, AH exhibited slightly superior functions compared to BB and ZM. However, in severe saline-alkaline soils, BB showed superior functional profiles compared to ZM and AH, particularly in categories such as metabolic pathways, biosynthesis of secondary metabolites, microbial metabolism in diverse environments, biosynthesis of amino acids, and carbon metabolism, all of which were stronger than those in AH. Given the enhanced sensitivity and substantial role of bacteria in plant adaptation to environmental stress, these findings shed light on how rhizosphere bacterial communities of different alfalfa varieties contribute to enhancing salt tolerance.

## Discussion

### The alfalfa rhizosphere microbial community is influenced and regulated by salinity and varieties.

Salinity is an important yet relatively understudied abiotic stress that that poses a significant threat to the growth and yield of alfalfa (Li and Brummer, 2012). However, different varieties exhibit varying responses to salt stress (Lei et al., 2018). As our results confirm, although increasing salinity levels significantly inhibit alfalfa growth and yield, salt-tolerant varieties often exhibit relatively better growth phenotypes and smaller yield losses compared to salt-sensitive varieties (**Figure 1**). Similar to other abiotic stresses like drought, salt stress typically leads to changes in the diversity or composition of the plant's microbial community. While these environmentally-induced changes may be directly driven by microbial responses, they are often indirectly initiated by plant reactions (Carvalhais et al., 2013). Plant responses can alter plant-microbe interactions through a series of interconnected mechanisms. This raises the question of whether differences in stress tolerance among species or varieties are also influenced and determined by the rhizosphere microbial regulation. To investigate whether the rhizosphere microbial composition of different salt-tolerant alfalfa varieties undergoes differential enrichment under environmental stress, potentially leading to variations in their salt tolerance, we conducted a study involving three alfalfa varieties with varying salt tolerance grown in different degrees of saline-alkali soil. Our primary focus was on characterizing the composition, differential taxa, and functional profiles of rhizosphere microbial communities among these different varieties in various degrees of saline-alkaline soils.

The key role of root-associated microorganisms in plant resistance to abiotic stressors has been underscored in recent research (Trivedi et al., 2021). Generally, it is observed that bacteria exhibit heightened sensitivity to abiotic stresses, resulting in more pronounced effects, while fungal community structures remain relatively stable (Thiem et al., 2018). As anticipated, our findings revealed a substantial reduction in the richness and diversity of rhizosphere bacteria in alfalfa with increasing soil salinity. In contrast, the impact on fungi was relatively minor (**Figure 4A**, **Supplementary Figure 3A**). This phenomenon could be attributed to the fact that the majority of bacteria were ill-suited for thriving in high-salinity environments (Yaish et al., 2016), whereas fungi have been shown to have greater stress resistance than bacteria (Gao et al., 2022). Furthermore, our study illuminated that microbial α-diversity does not follow a linear decline with rising salinity levels. This observation highlights that salinity is not the sole factor governing these variations. Notably, we have identified significant disparities in the responses of rhizosphere microbial diversity to salinity among different plant varieties. Indeed, plants with distinct genetic backgrounds manifest diverse physiological phenotypes and stress tolerances under salt stress conditions (Zelm et al., 2020). Previous research suggests that, through prolonged interactions with the environment, plants have evolved genotype-dependent mechanisms, reshaping their root-associated microbiota and enhancing their ability to cope with environmental stresses (Zhang et al., 2019; Yu et al., 2021). Our experimental results elucidated that, across soils characterized by varying degrees of salinity, the rhizosphere microbial diversity of the three alfalfa varieties showed substantial differences. Particularly notable was the pronounced impact on the salt-sensitive variety AH, which showed significantly lower bacterial diversity and richness, even in moderately saline-alkaline soils (**Figure 4A**). This underscores the critical role of plant variety in influencing microbial communities. Additionally, our analysis of β-diversity revealed that salt-tolerant varieties exhibit more closely related bacterial communities across soils with varying salinity levels. This suggests greater stability in composition and structure with relatively minor salinity-induced effects. In contrast, salt-sensitive varieties showed dissimilar bacterial communities among soils with different salinity levels (**Figure 4B**). These observations were further substantiated in our analysis of their correlations with various soil physicochemical factors. Salt-tolerant varieties exhibited no significant correlations between their rhizosphere bacteria and factors such as pH, salt content, and electrical conductivity, indicative of minimal influence. Conversely, salt-sensitive varieties exhibited a pronounced impact on their rhizosphere bacteria in response to factors such as pH, salt content, and electrical conductivity. While previous studies have suggested that, in certain high-salinity soil environments, plant genotype plays a smaller role in shaping rhizosphere bacterial communities compared to soil salinity (Li et al., 2013; Borruso et al., 2014), our results, along with findings in Arabidopsis (Ponsford et al., 2022), grapevine (Berlanas et al., 2019), maize (Aira et al., 2010), soybean (Liu et al., 2019), tomato (Cheng et al., 2020), and switchgrass (Liu et al., 2021), underscore the substantial influence of host plant genotype on the formation of rhizosphere communities.

In conclusion, our findings provide compelling evidence for the pivotal role of salinity in shaping the composition and structure of alfalfa rhizosphere microbiota. Furthermore, the abundance of relevant bacterial genera appears to be influenced and regulated by plant variety (genotype). Across soils characterized by varying degrees of salinity and alkalinity, different salt-tolerant alfalfa varieties exhibit significant disparities in rhizosphere microbiota, particularly concerning the abundance of specific bacterial genera.

### The differential composition of dominant rhizosphere microbial communities in different salt-tolerant alfalfa varieties is a potential key factor underlying their differences in stress resistance

The host genotype serves as a significant driving force shaping the composition of rhizosphere microbial communities, with salt-tolerant genotypes capable of recruiting specific bacterial taxa in saline-alkali soil (Li H. et al., 2021; Liao et al., 2023). By comparing the differences in rhizosphere microbial communities among three alfalfa varieties in soils with varying degrees of saline-alkaline, we observed significant variations in both composition and abundance. As salinity increased, salt-tolerant BB and ZM varieties exhibited an increase in unique bacterial genera, whereas AH displayed a reduction in unique bacterial genera and an increase in pathogenic fungal genera (**Supplementary Figure 6**). Different alfalfa varieties have the capability to adapt and selectively recruit and enrich rhizosphere bacterial members to promote microbial activity, thereby enhancing their adaptability in variable soil environments (Dumbrell et al., 2010; Ofek-Lalzar et al., 2014). It is noteworthy that regardless of salinity levels and variety, *Bacillus* was consistently the most abundant bacterium significantly enriched in the rhizosphere of alfalfa in saline-alkali soils (**Figure 5B**). This observation underscores the high adaptability of *Bacillus* to saline environments and its affinity for plant roots, suggesting its potential to enhance plant adaptability to salt stress. Furthermore, in severe saline-alkali soils, we observed significantly higher abundances of *Bacillus* and *Ensifer* in the rhizosphere of the salt-tolerant BB variety compared to ZM and AH, along with elevated *Pseudomonas* abundance (**Figure 5B**, **Supplementary Figure 5A**). The role of *Bacillus* in enhancing plant salt tolerance has been widely documented (Woo et al., 2020; Xiong et al., 2020; Yasmin et al., 2020). Alfalfa, inherently possessing moderate salt tolerance (Bertrand et al., 2015), indirectly demonstrates its capability through the substantial enrichment of *Bacillus* in the rhizosphere. Clearly, the abundance of *Bacillus*, particularly specific species, appears to be associated with the adaptability of alfalfa varieties to salinity, as indicated by our results (**Figure 8C**). Notably, the rhizosphere of BB, which exhibits superior salt tolerance, seemed to harbor a greater abundance of *Bacillus* compared to AH. *Ensifer*, previously identified in leguminous plants from saline-alkali soils, plays a unique role in nodulation, nitrogen fixation, and soil remediation in agriculture (Dang et al., 2019). Earlier studies have suggested that certain species of *Ensifer* (Bianco and Defez, 2009) and *Bacillus* (Xiong et al., 2020) can enhance plant salt tolerance. Our results further revealed that BB harbors a dominant bacterial genus, *Pseudomonas*. In fact, recent research has shown that *Pseudomonas* can maintain plant membrane integrity and alleviate salt-induced cell death in plants (Jha and Subramanian, 2015). Moreover, most species of *Pseudomonas* have the capacity to ameliorate salt stress in plants by producing stress-alleviating metabolites such as extracellular polysaccharides, gibberellins, ACC deaminase, and indoleacetic acid (Kumar et al., 2019; Etesami and Bernard, 2020). This underscores the pivotal role of the dominant core bacterial community, possibly a key factor contributing to the enhanced salt tolerance of BB. This is consistent with the findings of Yuan et al. (Yuan et al., 2016), who discovered that the core rhizosphere bacterium *Pseudomonas* in halophytes can enhance the salt tolerance of non-halophytic plants. Additionally, we observed marked inter-varietal differences in the response of these bacterial genera to varying levels of salinity stress, with a more similar trend in abundance among them in salt-tolerant varieties, unlike salt-sensitive varieties (**Figure 8B**).

Interestingly, we observed that, starting from moderate salinity levels, a significant enrichment of well-known pathogenic fungi, such as *Fusarium* and *Cladosporium* (Cosseboom and Hu, 2023), occurs in the rhizosphere of AH, and their relative abundance was significantly higher than that of BB and ZM (**Figures 5D**, **9A, B**, **Supplementary Figure 5A**). Furthermore, we identified a specific enrichment of highly pathogenic *Rhizoctonia* (Li C. et al., 2021) in the rhizosphere of AH (**Figure 9C**). These fungi are known key contributors to alfalfa root rot disease. This suggests that salt-sensitive varieties are more susceptible to soil-borne diseases. However, it is noteworthy that in the rhizosphere of BB, we found a specific enrichment of *Beauveria* at a higher abundance. This fungus has been extensively documented and employed as a biocontrol agent. *Beauveria* can form symbiotic relationships with plants and facilitate the transfer of soil nitrogen to the host, positively influencing plant growth and stress resistance (Behie and Bidochka, 2014). This may also be a key factor contributing to BB's enhanced salt tolerance. In summary, these results indicate that different alfalfa varieties can recruit distinct microbial communities to establish specific rhizosphere microbiomes, contributing to their growth, stress resilience, and overall health.

### Differences in the metabolic functions of rhizosphere bacterial communities in alfalfa determine the adaptability of different salt-tolerant varieties under salinity stress conditions

Differences in the rhizosphere microbiota among different alfalfa varieties ultimately manifest in their functional contributions. Current research suggests that bacteria play a more significant role than fungi in aiding plants to enhance their tolerance to environmental stress. To uncover how they assist alfalfa in improving salt tolerance, we conducted functional predictions on the rhizosphere bacterial communities of different alfalfa varieties in saline-alkaline soils. We observed that the rhizosphere bacteria of salt-tolerant varieties exhibited greater activity in various metabolic pathways, such as biosynthesis of secondary metabolites, carbon metabolism, and amino acid biosynthesis (**Figure 10**). It is well-known that microorganisms require carbon sources for growth, and increased carbon sources lead to heightened carbon metabolism and microbial activity. One essential function of PGPB is the production of various secondary metabolites like IAA, ACC, etc., which interact with the host, maintain osmotic balance, and enhance host stress tolerance (Ali and Khan, 2021). Therefore, we speculate that salt-tolerant alfalfa may provide more carbon sources to enrich a more effective population of PGPB in the rhizosphere, helping to alleviate stress. We also found that salt-tolerant alfalfa had a stronger amino acid biosynthesis pathway in the rhizosphere than salt-sensitive varieties. Amino acids can serve as carbon or nitrogen sources for rhizosphere bacterial proliferation under salt stress conditions (Sivolodskii, 2009) and generate abundant glutamate or aspartate as nitrogen sources for Pseudomonas proliferation (Köhler et al., 2000; Li and Lu, 2018). Pseudomonas can utilize tryptophan as an exogenous precursor to produce IAA (Ilangumaran et al., 2021), potentially promoting alfalfa root development. ABC transporters in bacteria can absorb substrates such as sugars, amino acids, metal ions, iron chelators, and vitamin B-12 to support their survival. Additionally, they are involved in bacterial material efflux, such as bacterial surface components (lipopolysaccharides, capsular polysaccharides), and siderophores (Davidson and Chen, 2004). Interestingly, our results showed that salt-tolerant varieties had a relatively higher abundance of ABC transporters in their rhizosphere bacterial communities, which could be a significant factor contributing to their increased salt tolerance. In conclusion, the rhizosphere bacterial communities of different salt-tolerant alfalfa varieties are closely related to their stress resistance. We are aware that the plant-microbe interactions are mediated through rhizosphere secretions, implying the presence of a regulatory process involving "gene expression-rhizosphere secretions-rhizosphere microbiota" to enhance plant stress tolerance (Hu et al., 2018; He et al., 2021; He et al., 2022). However, the detailed molecular mechanisms through which alfalfa utilizes rhizosphere microbiota to enhance abiotic stress tolerance require further in-depth exploration and validation.

## Data availability statement

The datasets presented in this study can be found in online repositories. The names of the repository/repositories and accession number(s) can be found below: https://www.ncbi.nlm.nih.gov/, PRJNA1028542.

## Author contributions

WF: Conceptualization, Formal analysis, Visualization, Writing – original draft, Writing – review & editing. YX: Data curation, Investigation, Writing – original draft, Writing – review & editing. JD: Formal analysis, Investigation, Writing – original draft, Writing – review & editing. JX: Investigation, Validation, Writing – original draft, Writing – review & editing. FT: Writing – review & editing, Resources, Visualization, Writing – original draft. FS: Funding acquisition, Writing – review & editing, Project administration, Writing – original draft.

## Footnotes

1
https://github.com/OpenGene/fastp,

2
http://www.cbcb.umd.edu/software/flash.

3
http://rdp.cme.msu.edu/.

4
https://cloud.majorbio.com.
