# Supplementary material for: Variety-driven rhizosphere microbiome bestows differential salt tolerance to alfalfa for coping with salinity stress
Source: Front Plant Sci. 2023 Dec 11;14:1324333. doi: 10.3389/fpls.2023.1324333 (PMC10766110; doi:10.3389/fpls.2023.1324333)
Supplement: Supplementary file 1 [file DataSheet_1.pdf]

Supplementary Material

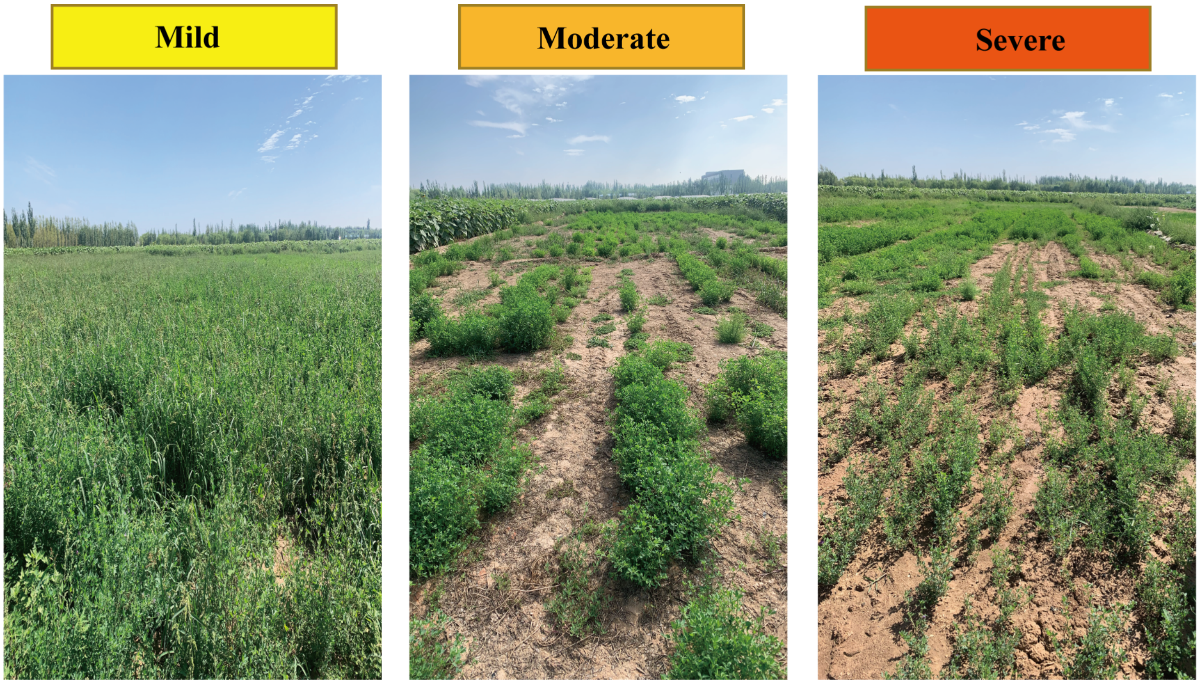

Supplementary Figure 1. Growth performance of alfalfa in soils with varied degrees of saline-alkali soil.

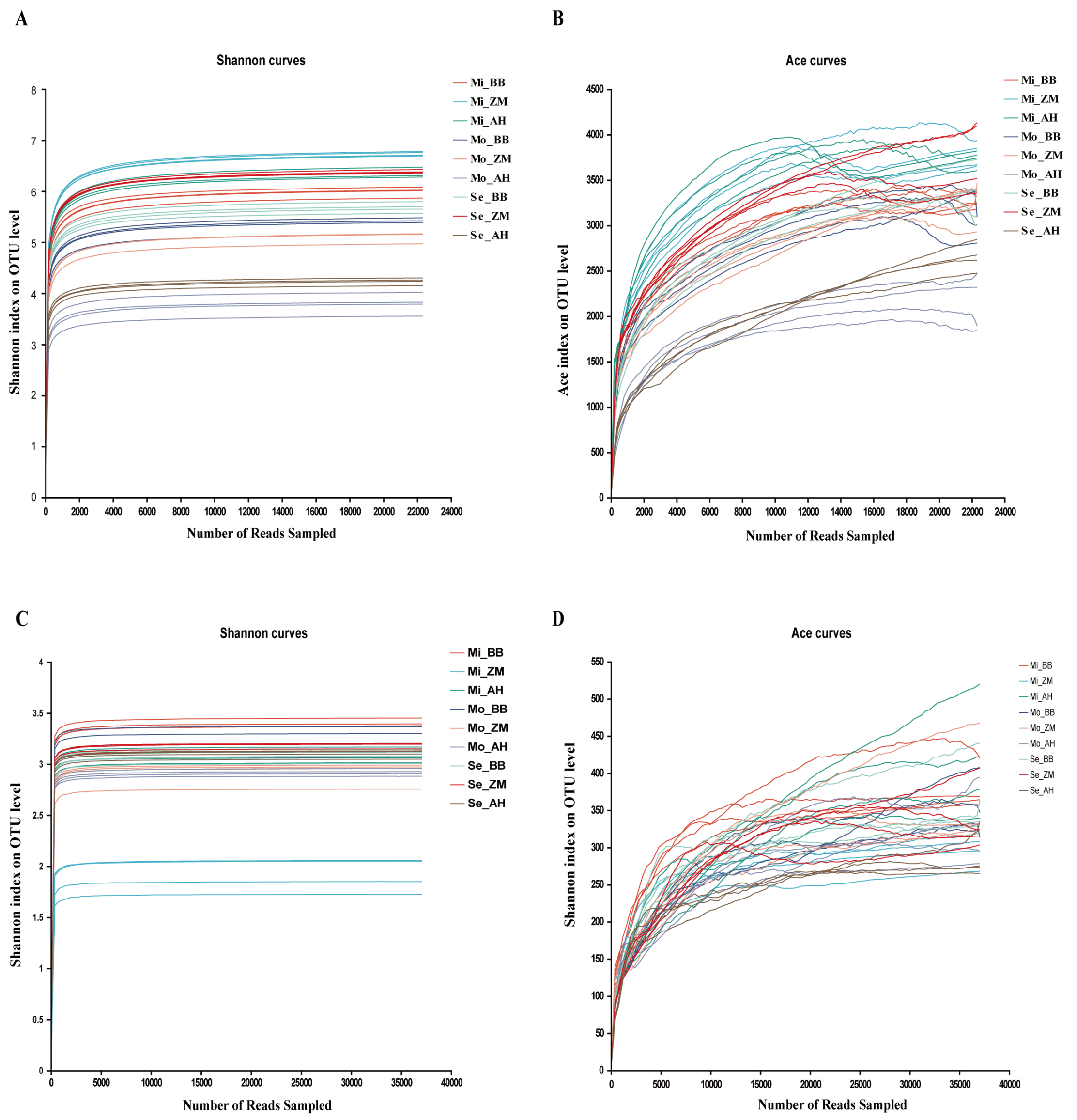

Supplementary Figure 2. Rarefaction curves. **(A)** Rarefaction curve of bacterial Shannon index, **(B)** Rarefaction curve of bacterial Ace index, **(C)** Rarefaction curve of fungal Shannon index, **(D)** Rarefaction curve of fungal Ace index.

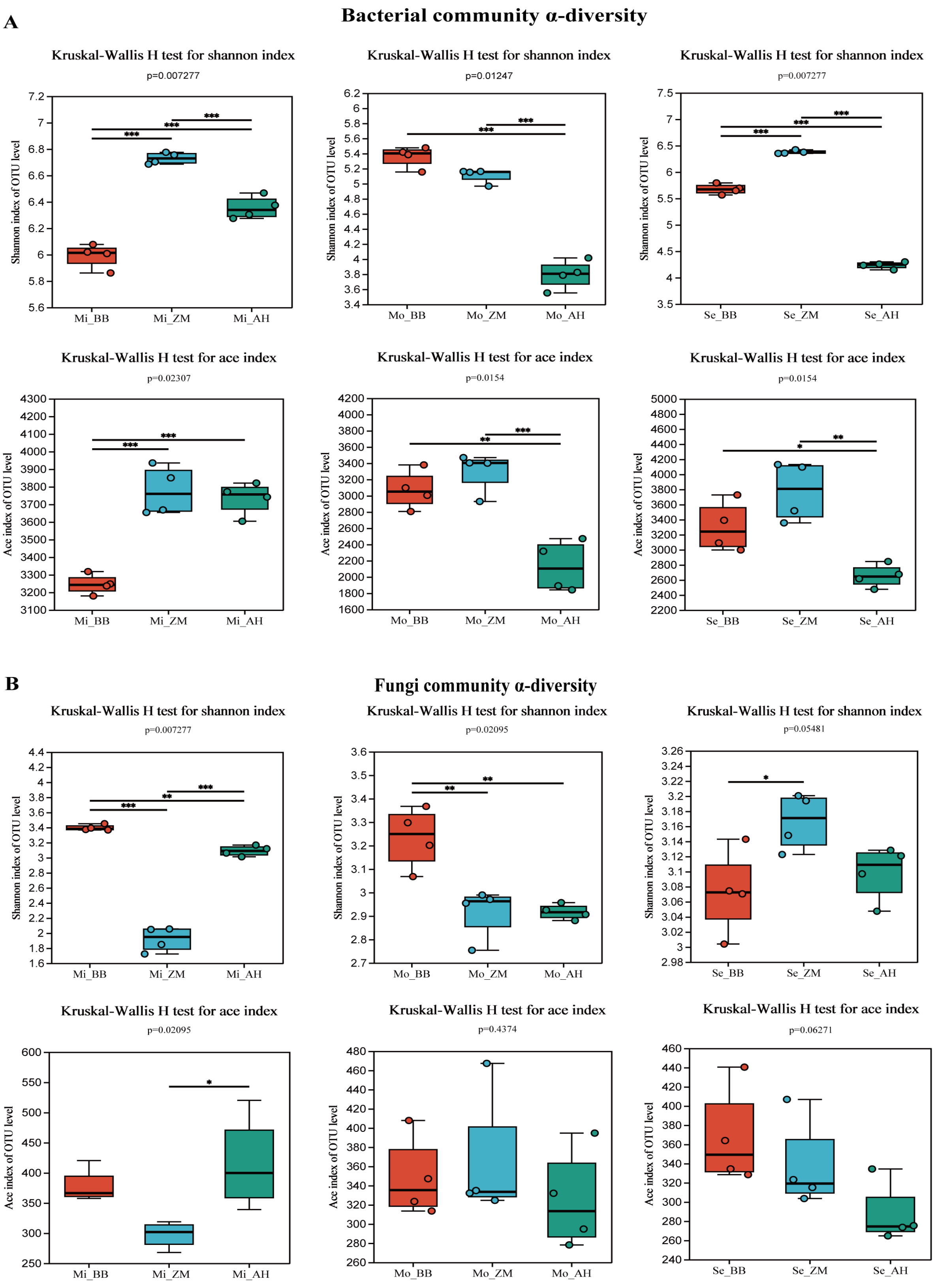

Supplementary Figure 3. Analysis of inter-group differences in Shannon and Ace indices of bacteria and fungi. **(A)** Differential analysis of bacterial Shannon and Ace indices, **(B)** Differential analysis of fungal Shannon and Ace indices.

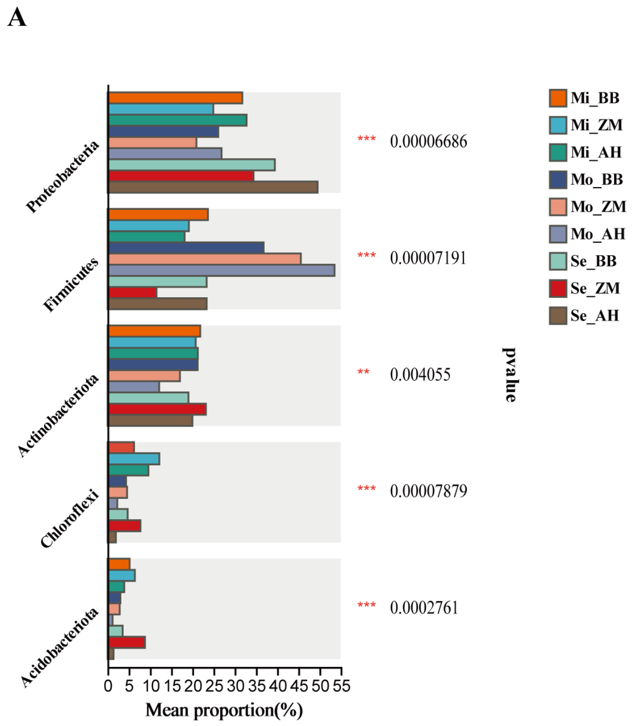

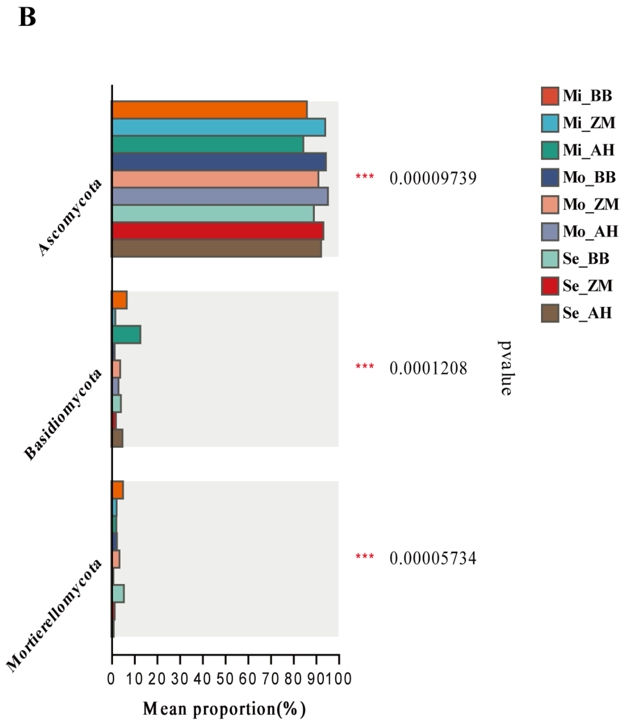

Supplementary Figure 4. Differential significance testing of variations in the main species composition of rhizosphere microbial communities at the phylum level among different alfalfa varieties in different degrees of saline-alkali soil. **(A)** Differential testing at the bacterial phylum level, **(B)** Differential testing at the fungal phylum level.

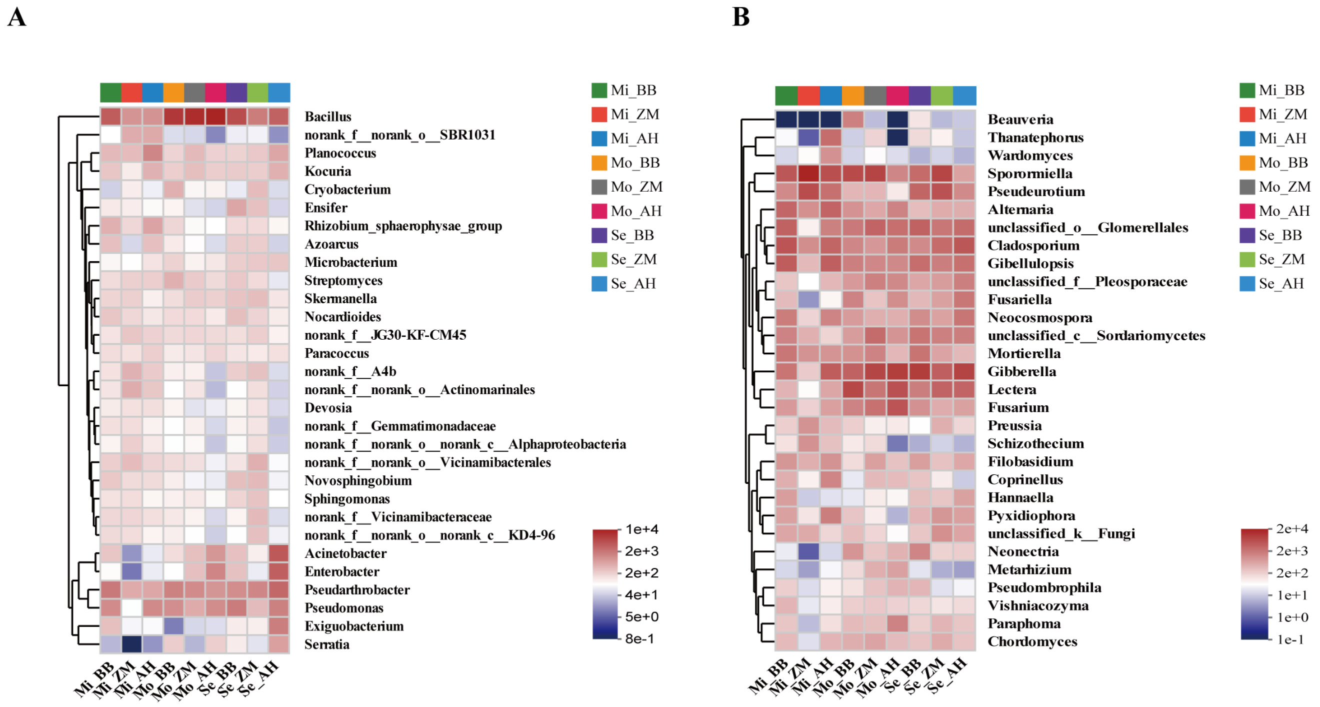

Supplementary Figure 5. A heat map clustering shows the average relative abundance of the top 30 genera of all samples, **(A)** The top 30 genera of bacteria, **(B)** The top 30 genera of fungi.

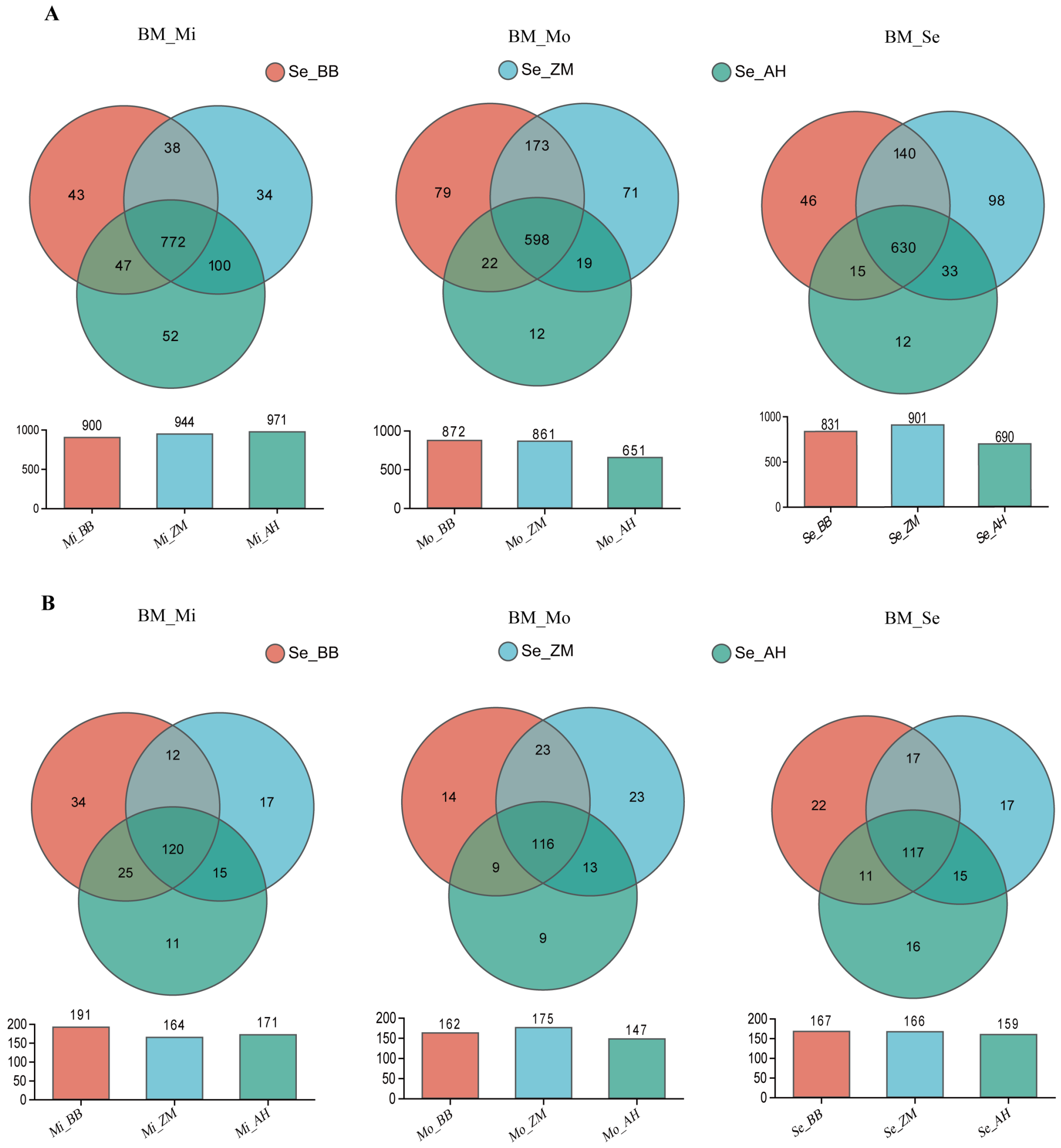

Supplementary Figure 6. Visualization of common and unique microbial genera in the rhizosphere of different varieties in saline-alkaline soil. **(A)** Bacteria, **(B)** Fungi.

Table S1. Bacteria and fungi α-diversity index

Sample group	Bacteria				Fungi			
	Shannon	Simpson	Ace	Chao1	Shannon	Simpson	Ace	Chao1
Mi_BB	5.99	0.01	3245.21	3173.65	3.40	0.06	377.56	371.52
Mi_ZM	6.73	0	3776.76	3696.61	1.92	0.36	297.58	297.92
Mi_AH	6.35	0.01	3734.2	3650.07	3.09	0.08	414.58	341.95
Mo_BB	5.36	0.02	3071.4	2947.03	3.23	0.08	347.94	342.67
Mo_ZM	5.11	0.05	3300.63	2898.17	2.92	0.12	364.67	327.69
Mo_AH	3.79	0.15	2129.57	1919.4	2.92	0.11	324.85	289.54
Se_BB	5.67	0.01	3300.95	2961.7	3.07	0.10	366.80	331.27
Se_ZM	6.38	0.01	3774.5	3458.11	3.17	0.08	337.15	324.99
Se_AH	4.23	0.05	2651.97	2114.66	3.10	0.09	286.95	272.78
